# A diverse ancestrally-matched reference panel increases genotype imputation accuracy in a underrepresented population

**DOI:** 10.1038/s41598-023-39429-3

**Published:** 2023-07-31

**Authors:** John Mauleekoonphairoj, Sissades Tongsima, Apichai Khongphatthanayothin, Sean J. Jurgens, Dominic S. Zimmerman, Boosamas Sutjaporn, Pharawee Wandee, Connie R. Bezzina, Koonlawee Nademanee, Yong Poovorawan

**Affiliations:** 1 Center of Excellence in Arrhythmia Research, Department of Medicine, Faculty of Medicine, Chulalongkorn University, Bangkok, Thailand; 2 Interdisciplinary Program of Biomedical Sciences, Graduate School, Chulalongkorn University, Bangkok, Thailand; 3 National Biobank of Thailand, National Science and Technology Development Agency, Pathum Thani, Thailand; 4 Division of Cardiology, Department of Pediatrics, Faculty of Medicine, Chulalongkorn University, Bangkok, Thailand; 5 Bangkok Hospital, Bangkok, Thailand; 6 Heart Center, Department of Experimental Cardiology, Amsterdam Cardiovascular Sciences, Amsterdam University, Medical Centre, University of Amsterdam, Amsterdam, The Netherlands; 7 Cardiovascular Disease Initiative, Broad Institute of MIT and Harvard, Cambridge, MA USA; 8 Pacific Rim Electrophysiology Research Institute, Bumrungrad International Hospital, Bangkok, Thailand; 9 Center of Excellence in Clinical Virology, Faculty of Medicine, Chulalongkorn University, Bangkok, Thailand

**Keywords:** Genomics, Haplotypes, Genetic variation

## Abstract

Variant imputation, a common practice in genome-wide association studies, relies on reference panels to infer unobserved genotypes. Multiple public reference panels are currently available with variations in size, sequencing depth, and represented populations. Currently, limited data exist regarding the performance of public reference panels when used in an imputation of populations underrepresented in the reference panel. Here, we compare the performance of various public reference panels: 1000 Genomes Project, Haplotype Reference Consortium, GenomeAsia 100 K, and the recent Trans-Omics for Precision Medicine (TOPMed) program, when used in an imputation of samples from the Thai population. Genotype yields were assessed, and imputation accuracies were examined by comparison with high-depth whole genome sequencing data of the same sample. We found that imputation using the TOPMed panel yielded the largest number of variants (~ 271 million). Despite being the smallest in size, GenomeAsia 100 K achieved the best imputation accuracy with a median genotype concordance rate of 0.97. For rare variants, GenomeAsia 100 K also offered the best accuracy, although rare variants were less accurately imputable than common variants (30.3% reduction in concordance rates). The high accuracy observed when using GenomeAsia 100 K is likely attributable to the diverse representation of populations genetically similar to the study cohort emphasizing the benefits of sequencing populations classically underrepresented in human genomics.

## Introduction

Variant imputation has become a mainstay in contemporary genome-wide association studies (GWAS), as the increased exploration and testing of unobserved genotypes improves statistical power^1^. Imputation uses haplotype information from a reference panel to infer genetic variation not typed, or typed inaccurately, by genotyping arrays, thereby correcting some genotyping errors and vastly enhancing genome coverage. The performance of imputation therefore relies heavily on the specific reference panel used.

The advent of next-generation sequencing has led to an increase in whole genome sequencing (WGS) availability, enabling the construction of high-density reference panels. While initially reference panels could accurately infer variants with minor allele frequencies (MAFs) ≥ 5%, the increased size and sequencing coverage of recent high-density panels has enabled imputation down to low-frequency, 5% > MAF ≥ 1%, and rare, MAF < 1%, variants^2–4^. This has allowed examination of the human genome at a finer resolution, leading to identification of thousands of novel associations in GWAS^5–7^.

A wide range of high-density public reference panels exists with varying sizes, sequencing coverages, and represented populations^1^. These public reference panels include the 1000 Genomes Project phase 3 (1000G), the Haplotype Reference Consortium (HRC), the GenomeAsia 100 K project (GenomeAsia), and the Trans-Omics for Precision Medicine (TOPMed) program. 1000G comprises 2504 ancestrally diverse individuals from 26 global populations^8, 9^. HRC covers 32,488 human genomes by combining WGS data from over 20 different studies including 1000G. WGS data from HRC have sequencing coverage of 4× to 8× and are predominantly of European descent^10^. GenomeAsia was constructed to address the underrepresentation of Asian populations in the preceding reference panels. GenomeAsia contains WGS data on 1739 individuals from over 219 populations across Asia, with high depth coverage (~ 36×)^11^. In their most recent release, TOPMed contains WGS of 97,256 individuals publicly available for imputation. TOPMed’s WGS data are high-depth coverage (~ 38×) including individuals from diverse ancestral backgrounds^12^.

Previous studies have demonstrated strong variations in imputation performance when common reference panels were applied to different populations^13, 14^. For example, imputation using HRC offered better accuracy among European populations than among the Han-Chinese population^14^. There are limited data regarding imputation performance when public reference panels are used in populations not widely represented in the reference. In turn, this causes difficulties in the reference selection, in understanding the limitations associated with each reference panel, and created challenges when performing genomic research in populations that are underrepresented. To our knowledge, the Thai population is not represented in any current public reference panel except for GenomeAsia (n = 2), and therefore, issues relating to imputation accuracy and panel selection are particularly important to genetic studies in this population. Here, we compare genotyping imputation of Illumina Global Screening Array (GSA) among Thai individuals using four different high-density reference panels (1000G, HRC, GenomeAsia, and TOPMed). Genotype yields and imputation accuracies at varying minor allele frequencies (MAF) were accessed. Lastly, the effect of reference panels selection on an association analysis and ability to impute rare disease-causing variants were examined.

## Results

### Genotype yield and confidence level

Four different public reference panels (1000G, HRC, GenomeAsia, and TOPMed) were used to impute genotyping array data in 412 Thai samples from the Southeast Asian Brugada syndrome cohort. The number of variant sites obtained varied when different reference panels were used. The largest number of variants (271 million [M]) was achieved when using TOPMed as a reference (Table 1). TOPMed reference resulted in 6 × more variant sites than 1000G (43.8 M), 7 × more than HRC (39.1 M), and 13 × more than GenomeAsia (21.5 M). In terms of insertion/deletions (INDEL), imputation using TOPMed resulted in 20.9 M INDELs, while 1000G resulted in 3.23 M. Due to the lack of INDEL data in HRC and GenomeAsia, INDELs could not be inferred when these two panels were utilized as imputation reference panels.

**Table 1:** Number of imputed variants at different imputation confidence levels according to Minimac-R^2^ cut-offs.

R^2^	Number of variant sites after imputation in millions (M)								
	GenomeAsia			1000G		TOPMed		HRC	
Cut-off	#SNP (M)	#INDEL	#SNP (M)		#INDEL (M)	#SNP (M)	#INDEL (M)	#SNP (M)	#INDEL
None	21.50	n/a	43.80		3.230	271.00	20.900	39.10	n/a
0.2	9.87	n/a	13.10		1.420	19.50	1.460	12.40	n/a
0.4	8.26	n/a	10.10		1.130	14.70	1.090	9.95	n/a
0.6	6.86	n/a	7.88		0.866	11.20	0.815	7.71	n/a
0.8	5.06	n/a	5.28		0.532	6.99	0.496	4.89	n/a

TOPMed also offered the largest number of imputed variant sites with high-confidence (R^2^ > 0.8) at 6.99 M when we examined variants imputation confidence levels using computed Minimac-R^2^ values (Table 1). Imputation using 1000G, GenomeAsia, and HRC obtained lower numbers of high-confidence variant sites (R^2^ > 0.8), at 5.28 M, 5.06 M, and 4.89 M, respectively. The number of variants reduced substantially when R^2^ cut-offs were applied. The largest reduction seen in the TOPMed-imputed data, with 92.8% of variant sites imputed using TOPMed are of low-confidence (R^2^ < 0.2). We examined the distribution of imputed variants over the range of 0.2–1.0 R^2^ (Supplementary Fig. 1). Imputation using GenomeAsia showed a large portion of variants with very high-confidence (15.7% at R^2^ of 0.9–1.0), while TOPMed showed the lowest number (1.42%; Table 1).

### Imputation accuracy

**Figure 1:**
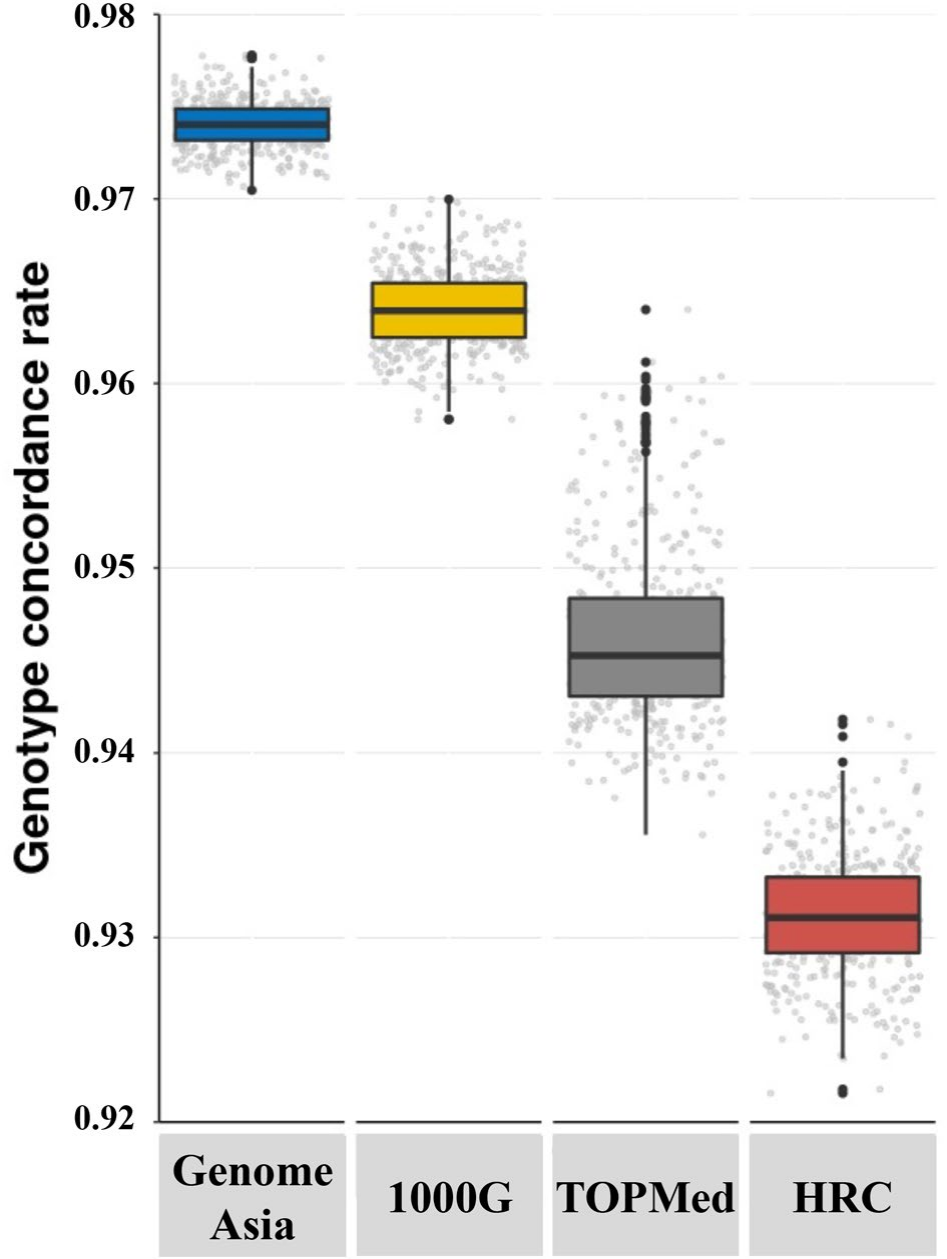
Imputation accuracy of chromosome 1 measured by GCR across 412 Thai individuals, using GenomeAsia, 1000G, TOPMed, and HRC reference panels. GCR was computed by comparison of imputed genotypes to validated genotypes from WGS. Data are presented as boxplots with distributions of sample GCR on the y-axis and imputation reference on the x-axis.

Imputation accuracy was examined used concordance between imputed genotypes and validation genotypes called from WGS to calculate Genotype Concordance Rate (GCR) for each sample. Overall, imputation using GenomeAsia achieved the highest accuracy with a cohort median GCR of 0.974 (Fig. 1; Q1–Q3 0.973–0.977). The median GCR values were substantially lower when using 1000G (0.964; Q1–Q3 0.962–0.965), TOPMed (0.945; Q1–Q3 0.943–0.948), and HRC (0.931; Q1–Q3 0.929–0.933) as a reference. Imputation accuracies were consistently high across samples within the cohort when using GenomeAsia as a reference (GCRs 0.970–0.978). Higher variations of GCR were found with TOPMed (0.935–0.963), where a small group of samples represented outliers with high GCR (Fig. 1). Examination of demographic data revealed that many individuals within this outlier group self-identified as Thai-Chinese (data not shown).

**Figure 2:**
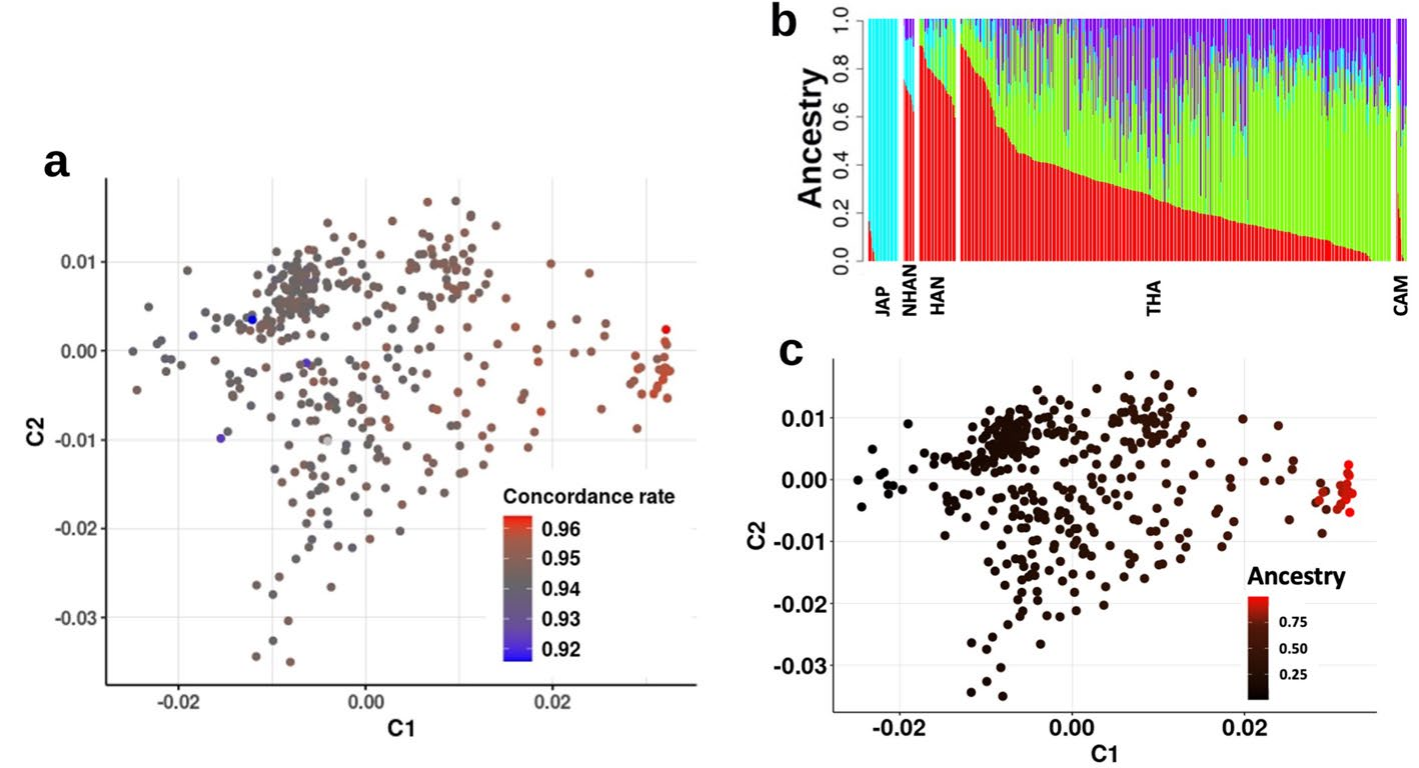
(**a**) Multidimensional scaling plot of 412 individuals coloured with GCR obtained when assessed genotypes imputed with TOPMed panel against genotypes from WGS. (**b**) Admixture plot of genome-wide genotype data of Thai (THA) and Japanese (JAP), Northern Han-Chinese (NHAN), Han-Chinese (HAN) and Cambodian (CAM) acquired from the Human Diversity Genome Project. (**c**) Multidimensional scaling plot coloured used the majority *Q* estimates found in Han-Chinese (HAN) from ADMIXTURE v. 1.3.

We investigated the effect of population structure within the Thai cohort on imputation accuracy when using the TOPMed panel. Using MDS analysis, we found that sample GCR values corresponded with the horizontal axis from the MDS analysis, and that samples with high GCR clustered together, separated from the other samples (Fig. 2a). Admixture analyses were performed to confirm that these samples represented the Thai-Chinese subpopulation, as suggested by the demographic data. Using North and South Han-Chinese genotype datasets acquired from the Human Diversity Genome Project, admixture analysis revealed that individuals within the high GCR cluster also had a higher degree of Han-Chinese admixture than the rest of the cohort (Fig. 2b,c).

When examined the effect of R^2^ cut-offs on imputation accuracy, imputation accuracy increased with more stringent R^2^ cut-off (Supplementary Fig. 2). At high-confidence imputed genotypes (R^2^ > 0.8), all samples achieved GCR above 0.967 regardless of reference panel used. TOPMed and HRC GCRs significantly improved with the median GCR approaching 0.974 and 0.973, respectively. GenomeAsia achieved the highest median GCR at 0.987.

### Imputation accuracy and allele frequency

**Figure 3:**
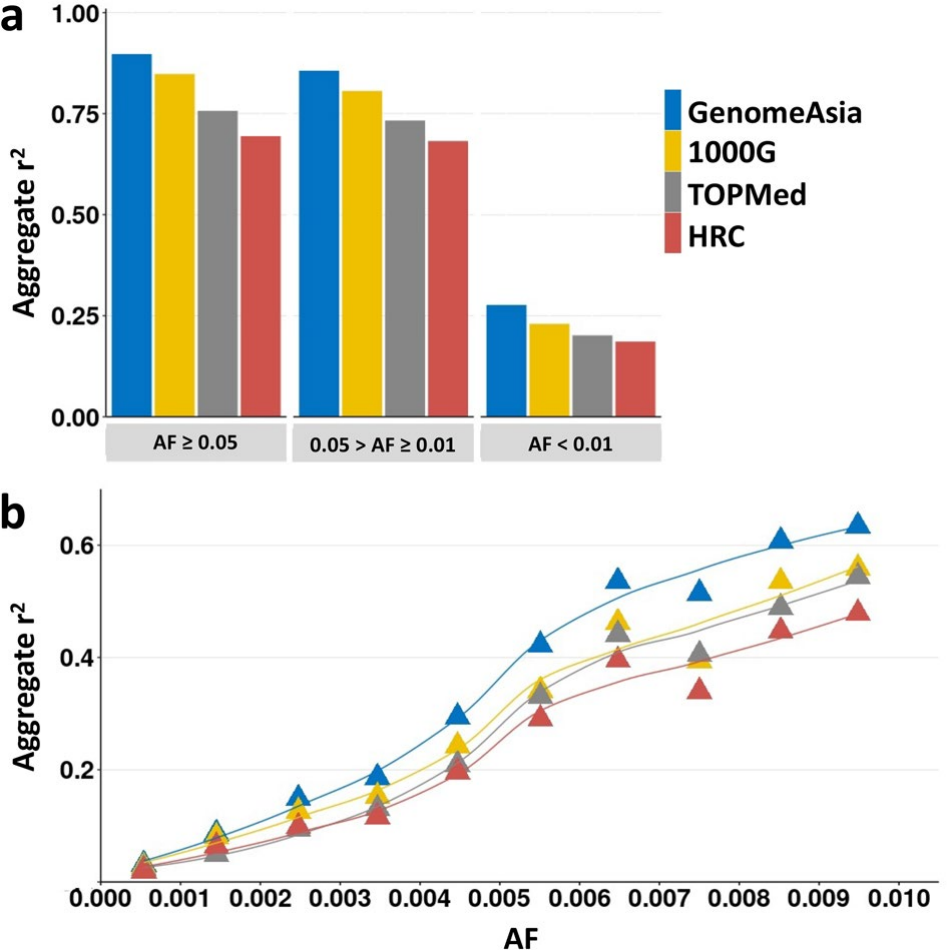
(**a**) Imputation accuracy of common (AF ≥ 0.05), low-frequency (0.05 > AF ≥ 0.01), and rare (AF < 0.01) variants. (**b**) Imputation accuracy of rare and ultra-rare variants at a finer resolution. Accuracies were measured using squared Pearson correlation between imputed and WGS variants. Variants r^2^ were aggregated into groups according to AF from gnomAD (version 2.1.1).

At different minor allele frequencies (MAFs), imputation using GenomeAsia offered better accuracy when compared to the other reference panels (Fig. 3a). Common variants (AF ≥ 0.05) and low-frequency variants (0.05 > AF ≥ 0.01) showed similar squared Pearson correlation patterns. Accuracy decreased considerably in the rare variants group (AF < 1%) for all four reference panels. For rare variants, GenomeAsia achieved the highest accuracy with squared Pearson correlation of 0.275, followed by 1000G (0.228), TOPMed (0.200), and HRC (0.184). Finer examination of rare and ultra-rare variant bins revealed that imputation accuracy decreased further with lower AF. GenomeAsia outperformed the other reference panels down to the 0.001 > AF ≥ 0 bin (Fig. 3b).

### Association study and imputation of protein-coding variants

We examined the impact of reference panels selection on an association analysis of genetic variants predisposing to Brugada Syndrome. GWASs were conducted on each of the four datasets that used different reference panels and genotypes called from WGS. Association statistics conducted using WGSs data showed 3 signals reached genome-wide significant threshold of 5 × 10^–8^; rs12634016 (*P* = 4.95511 × 10^–10^), rs7610489 (*P* = 1.12176 × 10^–9^) and rs1811852 (*P* = 4.83781 × 10^–8^) (Table 2). Dataset imputed with GenomeAsia also observed 3 signals in linkage disequilibrium with WGS’s lead SNP. When used 1000G, HRC, and TOPMed, only two signals reached genome-wide significant, rs12634016 and rs7610489. Signals at chromosomes 3 (rs12634016 and rs7610489) were imputed with high confidence (R2 > 0.8) for all reference panels used. Signals at chromosomes 6 were imputed with lower confidence with R^2^ ranging from 0.49 to 0.64.

**Table 2:** Signal associated with Brugada syndrome when different reference panels were used.

	WGS		GenomeAsia		1000G		HRC		TOPMed	
	Lead SNP	P value	Lead SNP	P value	Lead SNP	P value	Lead SNP	P value	Lead SNP	P value
1	3:38583025:G:A (rs12634016)	4.96E−10	3:38582571:A:G (rs6767797)	1.21E−10	3:38582571:A:G (rs6767797)	5.35 E−11	3:38582571:A:G (rs6767797)	1.67 E−09	3:38583025:G:A (rs12634016)	7.10 E−10
2	3:38806478:G:A (rs7610489)	1.12 E−09	3:38810525:C:T (rs56040630)	3.96 E−10	3:38810525:C:T (rs56040630)	6.26 E−10	3:38810525:C:T (rs56040630)	3.96 E−10	3:38814233:A:C (rs4420805)	9.83 E−10
3	6:126071575:G:C (rs1811852)	4.84 E−08	6:126115383:A:C (rs9388454)	3.34 E−09						

We further examined the ability of different high-density reference panels to infer rare disease-causing variants. Rare SCN5A protein-coding variants has been widely associated with Brugada syndrome. *SCN5A* coding variants inferred differs when different reference panels were used. TOPMed inferred 6 different variants, 2 variants were imputed when used 1000G and 1 variant when used HRC or GenomeAsia (Supplementary table 2). All variants have MAFs ranging from 0.0028 to 0.00002093 in gnomAD database and were imputed with R^2^ ranging from 0.34 to 0.71. The imputed variants were validated against used WGS. Imputation of rare SCN5A variants remain poor as all imputed rare SCN5A protein-coding variants found to be false positive.

## Discussion

In the present study, we evaluated the utility of four different public reference panels (1000G, GenomeAsia, HRC, and TOPMed) for variant imputation on Illumina Global Screening Array data of 412 Thai individuals, which are underrepresented in these references. We found that the choice of reference panel can strongly affect the breadth and accuracy of the resulting variant data. While TOPMed-based imputation resulted in the largest number of variants in our cohort, imputation using GenomeAsia achieved the greatest accuracy and lowest variability (GCR from 0.96 to 0.98). We further demonstrated that imputation accuracy can also be affected by the cohort population structure, as TOPMed imputation resulted in higher accuracy among samples with Chinese admixture. When considering the accuracy at different MAF groups, imputation using GenomeAsia outperformed other reference panels for rare and ultra-rare variants (0.002 > AF ≥ 0.001).

TOPMed represents an exceptionally large reference sample (N = 97,256). In concordance with previous studies, the larger reference size increases variant sites for imputation, including protein coding variants, that can be beneficial in further association analysis^15, 16^. Unfortunately, the larger TOPMed and HRC (N = 32,488) datasets, when used to impute our Thai cohort, achieved lower imputation accuracy than the smaller 1000G (N = 2504) or GenomeAsia (N = 1739) reference panels. A reduced performance of HRC has previously been described in non-European datasets, including those of Han-Chinese and African ancestry; here, it was suspected that the overrepresentation of European ancestry individuals in the HRC panel may cause bias during phasing and haplotype selection processes^14, 17^. While over 1184 East Asian individuals are represented in TOPMed, it only accounted for 1.22% of the total reference samples. Similar to HRC, the overrepresentation of populations with low genetic similarity to this study cohort in TOPMed may also be responsible for the low accuracy observed.

The high imputation accuracy of GenomeAsia may be attributable to its diverse representation of populations genetically similar to our study cohort. The GenomeAsia reference contains data from 219 population groups and 64 countries across Asia. Samples were collected from populations in the northeast, southeast and south Asia that are previously underrepresented in genetic studies. Indeed, a previous study demonstrated an improvement in imputation performance when additional populations were added to the reference^18^. Thailand is located at the center of mainland Southeast Asia with a high degree of genetic admixture from neighbouring countries through past migrational events^19^. While only 2 Thai WGS are represented in GenomeAsia, the diverse representation of genomes from neighboring countries likely provided a useful haplotype reference that benefited different subpopulations within our Thai cohort, leading to a higher accuracy throughout. In contrast, the diversity of Asian populations enrolled in the TOPMed study may not be as extensive with some Thai population subgroups underrepresented, as lower accuracies were observed in some samples within the cohort. The higher accuracy found in Thais with Han-Chinese admixture may reflect the high proportion of Han-Chinese ancestry represented in the East Asian population of the TOPMed database.

Although GenomeAsia yielded the best imputation accuracy for all AF bins, imputation accuracy strongly decreased with lower MAF as reported in other populations^20^. We found a 30.3% reduction in squared Pearson correlation of rare variants when compared to common variants. Several approaches have been proposed to improve imputation accuracy for rare variants. First and foremost, an increase in reference size strongly benefits rare variant imputation^21, 22^. As GenomeAsia currently has the smallest sample size of all four panels studied, an increase in Asian reference samples may vastly improve rare variant imputation accuracy. Secondly, using population-specific reference panels^4, 22, 23^. As costs decrease and sequencing becomes more widely accessible, WGS should enable the construction of a Thai population-specific reference panel in the near future.

We acknowledge several caveats and limitations of the present study. Imputation accuracy was not examined for all chromosomes, although similar results were obtained for chromosomes 1 and 21 (Supplementary Fig. 3). Evaluation of imputation accuracy was limited to WGS high-coverage regions. The accuracy of INDELs was not evaluated in this study, as this class of variation could only be obtained from imputation using TOPMed and 1000G reference panels.

In summary, our results demonstrate the importance and benefits of genetic similarity between reference and target datasets to achieve high imputation accuracy. Diverse representation of populations in the reference panel facilitates imputation of populations not represented well in the panel. For instance, GenomeAsia harbored a more diverse Asian population genetically similar to the Thai population, thereby outperforming other high-density reference panels in terms of imputation accuracy. Our findings emphasize the benefits of sequencing populations classically underrepresented in human genomics. As genomic studies are being perform in more diverse genetic ancestry, reference with diverse representation of populations would provide a crucial resource in studying these populations.

## Materials and method

In the present study, we enrolled 412 participants from the Southeast Asian Brugada syndrome cohort (ClinicalTrials.gov number, NCT04232787). The study was approved by the Institutional Review Board (IRB) of the Faculty of Medicine, Chulalongkorn University, Bangkok, Thailand (IRB No. 431/58). All methods were performed in accordance with relevant guidelines/regulations. Informed consent was obtained from all participants. All participants self-identified as Thai and were recruited from various regions of Thailand (Supplementary table 1).

Genome-wide genotyping and WGS were performed on all samples enrolled in this study. Genome-wide genotyping was done using the GSA platform, as previously described^24^. WGS with a mean coverage of 30× was carried out using the Illumina HiSeq X sequencer with paired-end reads of 150 bp, polymerase chain reaction-free, as previously described^25^. Alignments using the iSAAC aligner and variant caller using Starling version 2.4.7 were performed by Illumina Ltd, Cambridge, UK (Illumina Ltd, Cambridge, UK).

### Genotype imputation

Pre-imputation quality controls (QCs) were performed on genotyping array data following Scelsi et al. 2018 recommendations. PLINK (version 1.9) was used to exclude samples (1) with discordance between genetically inferred and self-reported sex, (2) genotype missingness > 0.05, and (3) with duplicates or first-degree relatives by using the *-rel-cutoff* command in PLINK (removing one member of each pair of samples with genomic relatedness > 0.5)^26^. Compatibility at variant level between genotyping array data and each of the reference panels was examined using the checking tools by W. Rayner (http://www.well.ox.ac.uk/~wrayner/tools/), to correct consistency of strand, alleles, positions, Ref/Alt assignments, and minor allele frequency differences. Imputation was performed on the Michigan Imputation Server (https://imputationserver.sph.umich.edu) using Eagle2 phasing and Minimac imputation. Based on the reference panels, 1000G, HRC, GenomeAsia, and TOPMed, four imputed genotype datasets were generated. Genotypes were extracted and counted using BCFtools (version 1.10.2). Minimac-R^2^ values, ranging from 0 (lowest confidence) to 1 (highest confidence), were used to reflect the imputation confidence for each imputed variant. Imputed variants were clustered according to five Minimac-R^2^ ranges: [0,0.2), [0.2,0.4), [0.4,0.6), [0.6,0.8), and [0.8,1].

### Evaluation of imputation accuracy

Imputation accuracy of the four imputed datasets that used the 1000G, HRC, GenomeAsia, and TOPMed reference panel were examined. Chromosome 1 variants from each of the imputed datasets were validated against high coverage genotypes called from WGS (among the same samples).

The WGS data underwent QC using Starling’s filtering criteria to filter out sites that have genotype conflicts with proximal indel calls, locus quality score < 30, locus quality score < 14 for heterozygous or homozygous variant, the fraction of basecalls at a site > 0.4, locus read evidence displays unbalanced phasing patterns, calls with a sample depth three times higher than the chromosomal mean, or genotype calls from variant callers not consistent with chromosome ploidy. Variant sites within the cohort with missingness > 0.10 or deviation from Hardy–Weinberg equilibrium (P value < 1 × 10^–6^) were excluded. Samples with > 0.05 genotype missingness were removed.

QCed WGS variant sites found in all four imputed genotyping datasets were selected for evaluation of imputation accuracy. Accuracy was measured in terms of genotype concordance rate (GCR) between the imputed and validating WGS data for each sample. The underlying GCR for each of the four reference panels was examined and visualized used ggplot2 package in R (version 3.6.3). Evaluation of imputation accuracy was further performed using chromosome 21 variants as validation.

### Imputation accuracy and allele frequencies

Imputation accuracy of variants at different allele frequencies (AFs) were examined used total AF from Genome Aggregation Database (gnomAD) version 2.1.1. The squared Pearson correlation between imputed and validating WGS variants was used to measure imputation accuracies. Variants were classified into AF bins according to gnomAD AFs. Variants were binned at 1, 0.05, and 0.01, to represent common, low-frequency, and rare variants, respectively. Finer examination of rare variants was performed following AF bins at 0.01, 0.009, 0.008, 0.007, 0.006, 0.005, 0.004, 0.003, 0.002, and 0.001. Square Pearson correlation were computed for each AF bin used GLIMPSE concordance tools^27^.

### Population structure and admixture analysis

The Thai cohort population structure was examined using a multidimensional scaling (MDS) method implemented in PLINK (version 1.9). Genotyping array data were pruned with parameters *-indep-pairwise 50 10 0.2*, leaving 135,661 markers. MDS was performed using *-mds-plot* function and visualized using R (version 3.6.3) to examine the presence of cohort population sub-structure. Chinese genetic admixture in the study cohort was examined used genotype dataset of 44 North and South Han-Chinese samples acquired from the Human Diversity Genome Project. Genetic admixture was estimated using ADMIXTURE software version 1.3 under the setting of K = 2^28^.

### Association analysis

The association study was performed using a frequentist association test as implemented in SNPTEST v.2.5.4 (open source, university of Oxford, Oxford, UK, https://mathgen.stats.ox.ac.uk/genetics_software/snptest/snptest.html). The genome-wide statistical significance threshold was set to P < 5 × 10^–8^.

## Supplementary Information

Supplementary Information 1.

Supplementary Information 2.

## Data Availability

The data that support the findings of this study are available from the National Research Council of Thailand, but restrictions apply to the availability of these data, which were used under license for the current study, and are not publicly available. Data are, however, available from the authors upon reasonable request and with permission of the National Research Council of Thailand.
