# Supplementary material for: A diverse ancestrally-matched reference panel increases genotype imputation accuracy in a underrepresented population
Source: Sci Rep. 2023 Jul 31;13:12360. doi: 10.1038/s41598-023-39429-3 (PMC10390539; doi:10.1038/s41598-023-39429-3)
Supplement: Supplementary file 1 — Supplementary Information 1. [file 41598_2023_39429_MOESM1_ESM.pdf]

Supplementary Information

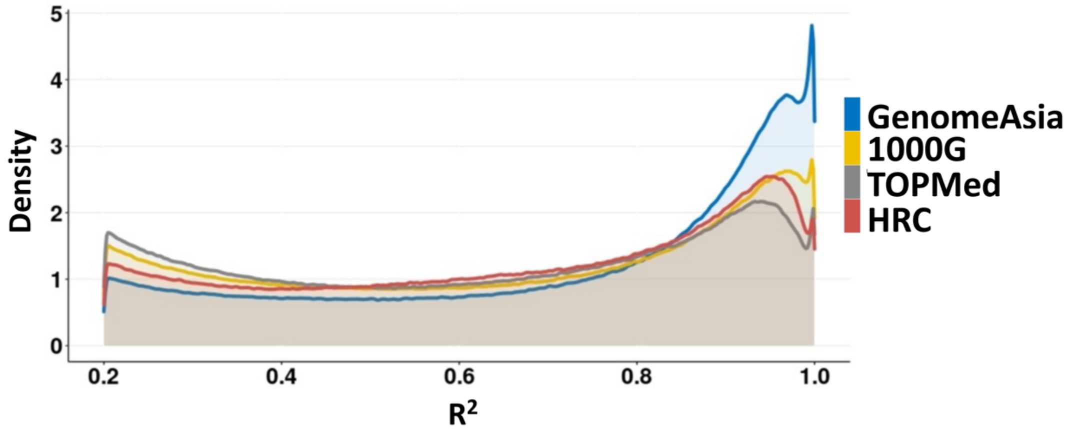

Supplementary Figure 1. Density plot of variants according to their Minimac R^2 values (ranging from 0.2 to 1.0) after imputed using GenomeAsia, 1000G, TOPMed, or HRC reference panel. The y-axis represents the density, while the x-axis represents the R^2 values.

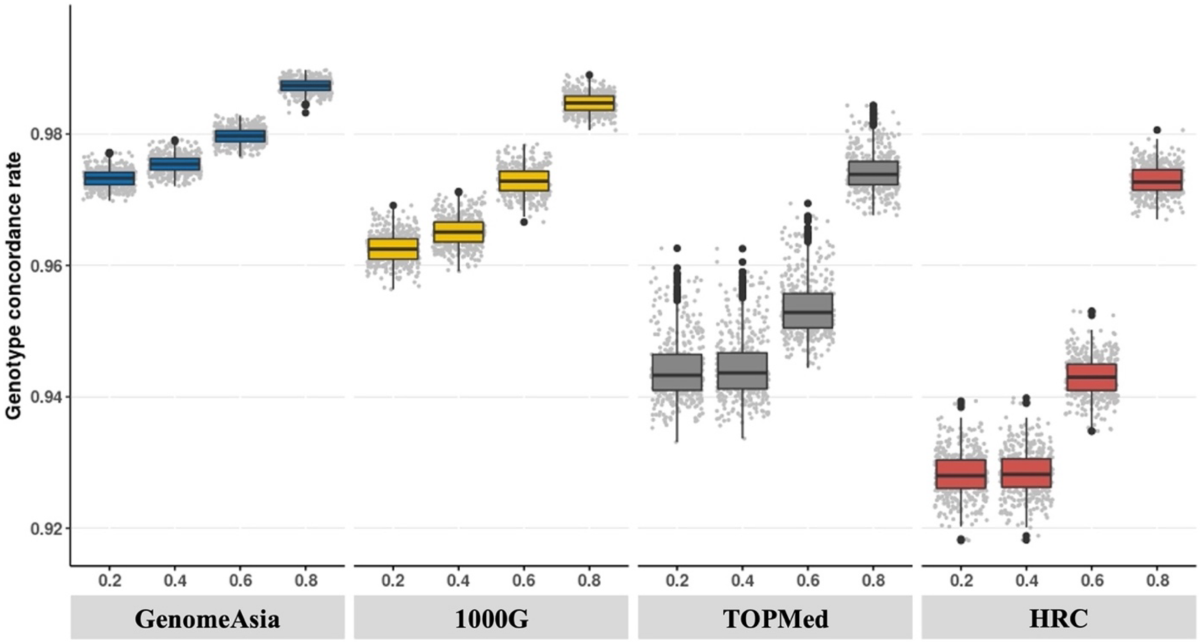

Supplementary Figure 2. Imputation accuracy of Thai cohort using the GenomeAsia, 1000G, TOPMed, and HRC reference panels, varying the R^2 cut-offs at 0.2, 0.4, 0.6, or 0.8. The imputation accuracies were evaluated using GCR. Data are represented using boxplots on the y-axis, with different R^2 cut-offs by reference panel shown on the x-axis.

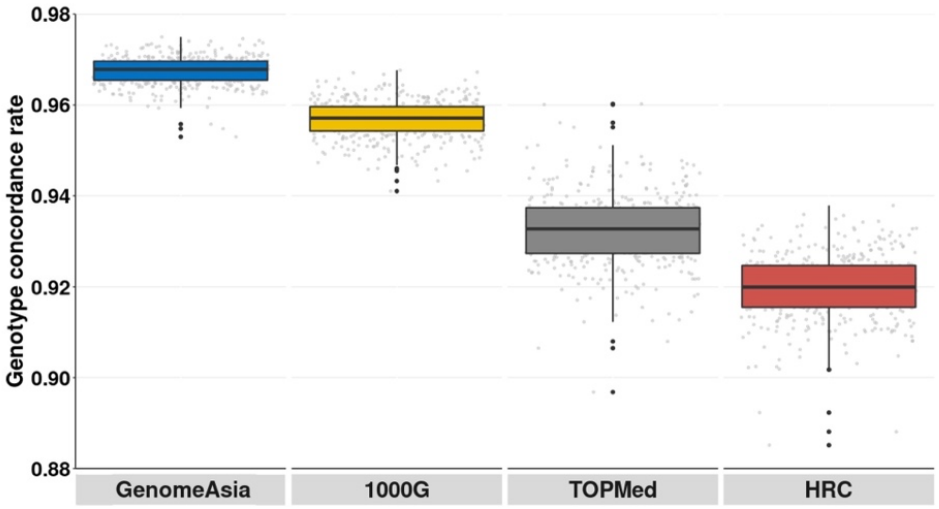

Supplementary Figure 3. Imputation accuracy of chromosomes 21 measured by GCR across 412 Thai individuals, using GenomeAsia, 1000G, TOPMed, and HRC reference panels. GCR was computed by comparison of imputed genotypes to validating genotypes from WGS. Data are presented as boxplots with distributions of sample GCR on the y-axis and imputation reference on the x-axis.
